# The nucleoside-diphosphate kinase NME3 associates with nephronophthisis proteins and is required for ciliary function during renal development

**DOI:** 10.1074/jbc.RA117.000847

**Published:** 2018-08-15

**Authors:** Sylvia Hoff, Daniel Epting, Nathalie Falk, Sophie Schroda, Daniela A. Braun, Jan Halbritter, Friedhelm Hildebrandt, Albrecht Kramer-Zucker, Carsten Bergmann, Gerd Walz, Soeren S. Lienkamp

**Affiliations:** From the ‡ Department of Medicine, Renal Division, Medical Center–University of Freiburg, Faculty of Medicine, University of Freiburg, 79106 Freiburg, Germany,; § Department of Medicine, Boston Children's Hospital, Harvard Medical School, Boston, Massachusetts 02115,; ¶ Center for Human Genetics, Bioscientia, 55218 Ingelheim, Germany, and; ‖ Center for Biological Signaling Studies (BIOSS), 79104 Freiburg, Germany

**Keywords:** primary cilium, kidney, DNA damage response, nucleoside/nucleotide metabolism, Xenopus, zebrafish, ciliopathies, nephronophthisis

## Abstract

Nephronophthisis (NPH) is an autosomal recessive renal disease leading to kidney failure in children and young adults. The protein products of the corresponding genes (NPHPs) are localized in primary cilia or their appendages. Only about 70% of affected individuals have a mutation in one of 100 renal ciliopathy genes, and no unifying pathogenic mechanism has been identified. Recently, some NPHPs, including NIMA-related kinase 8 (NEK8) and centrosomal protein 164 (CEP164), have been found to act in the DNA-damage response pathway and to contribute to genome stability. Here, we show that NME/NM23 nucleoside-diphosphate kinase 3 (NME3) that has recently been found to facilitate DNA-repair mechanisms binds to several NPHPs, including NEK8, CEP164, and ankyrin repeat and sterile α motif domain–containing 6 (ANKS6). Depletion of *nme3* in zebrafish and *Xenopus* resulted in typical ciliopathy-associated phenotypes, such as renal malformations and left-right asymmetry defects. We further found that endogenous NME3 localizes to the basal body and that it associates also with centrosomal proteins, such as NEK6, which regulates cell cycle arrest after DNA damage. The ciliopathy-typical manifestations of NME3 depletion in two vertebrate *in vivo* models, the biochemical association of NME3 with validated NPHPs, and its localization to the basal body reveal a role for NME3 in ciliary function. We conclude that mutations in the *NME3* gene may aggravate the ciliopathy phenotypes observed in humans.

## Introduction

Nephronophthisis (NPH)^4^ is an autosomal recessive kidney disease and is the main genetic cause of renal failure in pediatric patients. Typical clinical features are corticomedullary cysts and a urinary concentration defect (1–3). Many affected children also display extrarenal symptoms, including situs inversus, retinitis pigmentosa, hepatic fibrosis, and cerebellar dysplasia. All of these are typical manifestations of ciliopathy disorders, which include NPH, Meckel–Gruber syndrome, Joubert syndrome, and Bardet–Biedl syndrome (BBS) (1).

Causative mutations for NPH have been found in more than 19 genes so far. Their protein products are termed NPHPs, but no common motif or structural feature unites this group. However, most NPHPs are expressed in or near the primary cilium, which serves as a sensory organelle in most postmitotic cells (2, 4).

The precise function of most NPHPs in the cilium is not well understood. Detailed protein interaction mapping and high-resolution localization analysis allowed the NPHPs to be grouped into distinct “protein modules” that are likely to represent protein complexes (5–7). For example, NPHP1, -4, and -8 are found at the ciliary transition zone, which links the basal body and the ciliary axoneme and serves as a diffusion barrier (5). Some NPHPs like TTC21B (NPHP12), WDR19 (NPHP13), and IFT172 (NPHP17) are members of the intraflagellar transport (IFT) machinery (8–10). Other NPHPs such as CEP290 (NPHP6), CEP164 (NPHP15), and CEP83 (NPHP18) are localized on the basal body, which anchors the cilium within the cell (11–13). However, the localization of NPHPs into distinct subciliary zones is not mutually exclusive. For example, NEK8 and INVS have also been observed both in the proximal axoneme and at the basal body (5, 14, 15).

Despite the rapid discovery of novel disease-causing genes, about 30% of patients with NPH do not have a mutation in any of the known *NPHP* genes. The majority of mutations are found in *NPHP1* and account for ∼20% of total cases. With the exception of *NPHP1* and *NPHP4*, all other identified loci only account for less than 3% of total cases worldwide (16). This has made detection of novel disease genes increasingly difficult and demonstrates the need for testing of even larger patient cohorts. One of the main challenges remains in identifying the “missing NPHPs” that account for the large number of genetically undiagnosed cases. Novel functional protein partners of known NPHPs may shed new light on the pathogenesis of disease and might serve as candidates for screening genetically undiagnosed patients with NPH. Although the pathogenesis of ciliopathies is still elusive, it is increasingly recognized that the response to DNA-damage and replication stress is affected by mutations in some ciliopathy genes. The NPHPs NEK8 (NPHP9), SDCCAG8 (NPHP10; BBS16), ZNF423 (NPHP14; JBS19), and CEP164 (NPHP15) have all been linked to aberrant responses to DNA damage (17–20). SDCCAG8, ZNF423, and CEP164 are localized on nuclear foci positive for TIP60, known to activate ataxia telangiectasia mutated (ATM) at sites of DNA damage (18, 20).

In particular, NEK8 has been shown to be a critical component in the replication stress response. It promotes the progression and stability of the replication fork, thus preventing double-strand breaks during replication stress. Indeed, cystic kidneys of *Nek8* mutant mice (*jck*) show increased levels of γH2AX, a marker of DNA damage (19). NEK8 binds to components of the ATM- and Rad3-related (ATR) pathway and enables the replication fork protector RAD51 to assemble at sites of DNA repair (17).

The nucleoside-diphosphate kinase NME3 (NM23-H3; DR-NM23) belongs to a family of 10 nucleoside-diphosphate kinases (NDPKs, NMEs, or NM23), which catalyze the phosphorylation of nucleoside diphosphates (*e.g.* GDP) to nucleoside triphosphates (*e.g.* GTP) using ATP as an energy source (21). Recently, NME3 was found to directly bind to TIP60 and generate dNTPs at DNA-damage sites, thus supplying necessary nucleotides for subsequent repair (22).

Here, we uncover a surprising role for NME3 in cilia and show that NME3 physically interacts with NEK8, ANKS6, and other NPHPs of distinct NPHP modules. We demonstrate that Nme3 depletion in two vertebrate model organisms causes renal developmental defects and that Nme3 is required for determining left-right body axis specification and ciliogenesis. The detection of endogenous NME3 at the basal bodies of primary cilia and its physical interaction with ciliary components, including the centrosomal protein NEK6, further support a ciliary function. Because both NME3 and NEK6 are essential components of the DNA-damage response pathway, our data further strengthen the link between NPH and maintenance of genomic stability.

## Results

**Figure 1.**
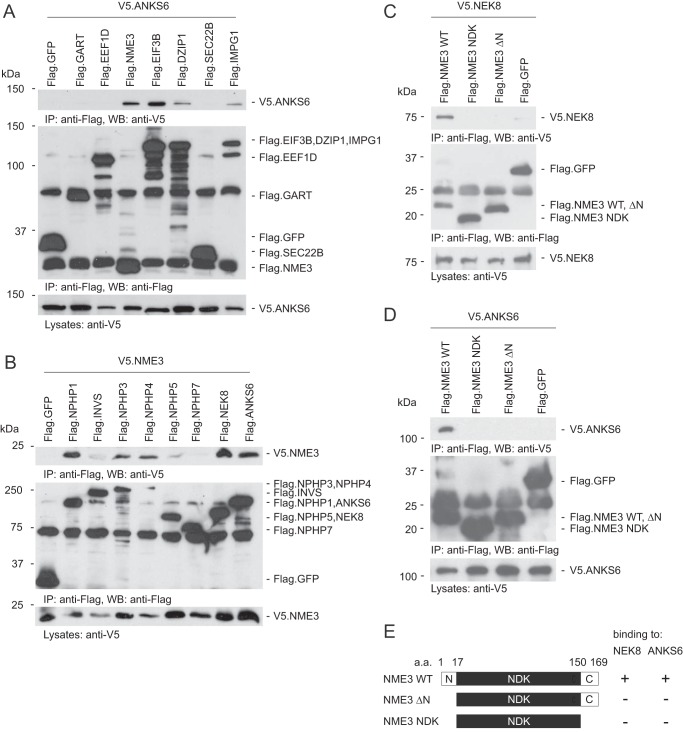
**NME3 interacts with NPHP1, -3, -4, NEK8, and ANKS6, and the N-terminal 17 amino acids are necessary for its interaction with ANKS6 and NEK8.** *A*, V5-tagged ANKS6 was coexpressed with different FLAG-tagged proteins in HEK293T cells. After immunoprecipitation with an anti-FLAG antibody, ANKS6 was detected in the precipitates of NME3, DZIP1, EIF3B, and IMPG1. It was not detected in the precipitate of GFP, which served as a negative control. *B*, transient overexpression of V5-tagged NME3 and FLAG-tagged NPHPs followed by immunoprecipitation with an anti-FLAG antibody. NME3 was present in precipitates of NPHP1, -3, -4, NEK8, and ANKS6. *C*, V5-tagged NEK8 was coexpressed with different mutant forms of FLAG-tagged NME3 in HEK293T cells. After immunoprecipitation with an anti-FLAG antibody, NEK8 was detected in the precipitate of WT NME3. Only a faint, nonspecific band was detected in the precipitate of GFP, which served as a negative control. *D*, transient overexpression of V5-tagged ANKS6 and different mutant forms of FLAG-tagged NME3 followed by immunoprecipitation with an anti-FLAG antibody. ANKS6 was only present in the precipitate of WT NME3. *E*, schematic of NME3 truncations used for immunoprecipitation experiments. NME3 contains a kinase domain (*NDK*). *a.a.*, amino acids; *IP*, immunoprecipitation; *WB*, Western blotting.

We previously identified ANKS6 as a novel NPHP (NPHP16) by screening for potential protein interactors of NEK8 (NPHP9) (6). When using NEK8 as bait in an affinity purification screen, we identified by MS analysis several additional binding partners. As NEK8 is a member of the ANKS6 module, the potential interactors were further validated by coimmunoprecipitation with ANKS6 in human embryonic kidney (HEK) 293T cells. Among the proteins identified in the NEK8 pulldown, which also bound to ANKS6, were the centriolar protein DZIP1 that has been linked to ciliogenesis in zebrafish and the BBSome (23, 24), interphotoreceptor matrix glycoprotein IMPG1, the translation elongation factor EIF3B, and the nucleoside-diphosphate kinase NME3 (Fig. 1*A*). The strongest interaction to ANKS6 was observed with EIF3B and NME3.

Members of the NME protein family (group II; NME5–9) are expressed in the axonemes of cilia and flagella (25). A knockout mouse for *Nme7* shows situs inversus (26, 27), and the loss of *NME5*, *-7*, or *-8* causes primary ciliary dyskinesia (28, 29). The RP2 protein (also known as NME10) is encoded by a gene that when mutated causes retinitis pigmentosa in humans (30). Because many members of the NME protein family are essential for ciliary function and their loss leads to various manifestations within the spectrum of ciliopathic disorders, we focused on NME3 in further analyses.

We first investigated whether NME3 could also function as a binding partner for other NPHPs. We observed that NME3 binds to NPHP3, NEK8, and ANKS6 but also to NPHP1 and NPHP4 in coimmunoprecipitation experiments (Fig. 1*B*). Fragment analysis demonstrated that the N-terminal 17 amino acids of NME3 are essential for the interaction with NEK8 and ANKS6 (Fig. 1, *C*, *D*, and *E*).

**Figure 2.**
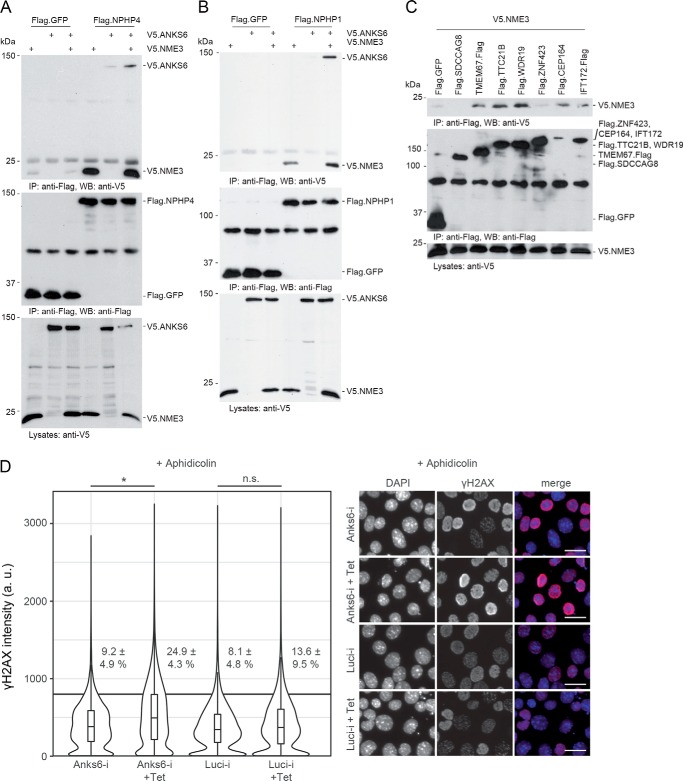
**NME3 functions as a link between the INVS, NPHP3, NEK8, ANKS6 module and the NPHP1, -4, -8 module.** *A*, FLAG-tagged NPHP4 was coexpressed with V5-tagged ANKS6 and NME3. After immunoprecipitation with an anti-FLAG antibody, NPHP4 was detected in the precipitate in which NME3 was coexpressed (*lane 6*). *B*, FLAG-tagged NPHP1 was coexpressed with V5-tagged ANKS6 and NME3. After immunoprecipitation with an anti-FLAG antibody, NPHP1 was detected in the precipitate in which NME3 was coexpressed (*lane 6*). *C*, NME3 showed physical interaction with TMEM67, TTC21B, WDR19, CEP164, and IFT172. *D*, IMCD3 cells were immunostained for γH2AX, and mean intensity measurements were performed by high-content screening microscopy for at least 3000 cells per condition (violin plots) in three replicates. Percentages and standard deviations of cells above a mean intensity cutoff at 800 absorbance units (*a.u.*) indicated by a *horizontal line* are given (*, *p* = 0.014; *n.s.*, not significant; *t* test). Representative images are shown on the *right. Scale bars*, 25 μm (*D*). *IP*, immunoprecipitation; *WB*, Western blotting; *Tet*, tetracycline; *Luci*, luciferase.

We previously showed that NPHP3, NEK8, and ANKS6 belong to a distinct NPHP module similar to NPHP1, NPHP4, and NPHP8 (6). Because NME3 interacts with members of both modules, we asked whether it acts as a link between these protein groups. Therefore, we tested whether the presence of NME3 facilitates binding of NPHP1/NPHP4 to ANKS6, two proteins that do not interact directly. Immunoprecipitation of either NPHP1 or NPHP4 revealed that coexpression of NME3 facilitated binding to ANKS6 (Fig. 2, *A* and *B*). These findings suggest that NME3 may stabilize the interactions between components of the two NPHP modules.

Next, we tested a number of recently identified and less well characterized NPHPs for interaction with NME3 (Fig. 2*C*). We detected binding of NME3 to TMEM67, TTC21B, WDR19, CEP164, and IFT172. IFT172, WDR19, and TTC21B are members of the IFTA system in cilia, whereas CEP164 is localized in the basal body (9, 12, 31). Interestingly, IFT172, WDR19, and TTC21B can also interact with ANKS6 (Fig. S1*A*), suggesting a closer link between the ANKS6 module proteins and the IFT machinery.

Because NME3 and several interacting NPHPs, including NEK8 and CEP164, have been linked to DNA-repair mechanisms (17–20, 22), we investigated a potential role for ANKS6 in the DNA-damage response pathway. Knockdown of Anks6 in an inner medullary collecting duct (IMCD) 3 cell line that expresses an inducible shRNA did not alter the intensity of γH2AX staining at baseline (Fig. S1*B*). However, aphidicolin-mediated replication stress led to significantly increased γH2AX signal intensity in Anks6 knockdown cells compared with the intensity in the noninduced condition or that in cells expressing a control shRNA (Fig. 2*D*, *Luci-i*).

**Figure 3.**
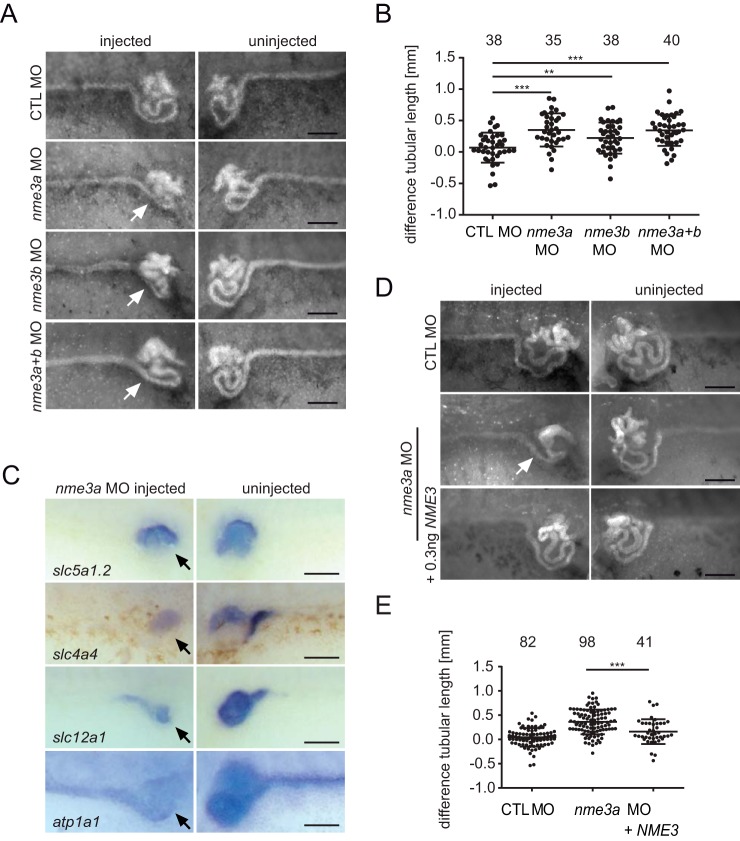
**Nme3-depleted *Xenopus* embryos exhibit pronephric malformations.** *A*, after unilateral injection of control (*CTL*) MO, *nme3a* MO, and/or *nme3b* MO, *Xenopus* embryos were stained with fluorescein-conjugated lectin to visualize the pronephric epithelia. The morphants showed a strong simplification of the proximal tubules on the *nme3* MO–injected side in contrast to the uninjected side (*white arrows*). *Scale bars*, 200 μm. *B*, differences in tubular length between the uninjected and injected sides were measured and calculated (**, *p* = 0.008; ***, *p* < 0.001; *t* test; *error bars* represent S.D.). *C*, after unilateral injection of *nme3a* MO, WISH for different pronephric segment markers was performed. The expression of all markers investigated was reduced on the *nme3a* MO–injected side (*black arrows*). *Scale bars*, 50 μm. *D*, representative pictures of pronephric tubules stained by fluorescein-conjugated lectin. The simplification of the pronephric tubule observed upon *nme3*a depletion (*white arrow*) was partially rescued by coinjection of human *NME3* RNA. *Scale bars*, 200 μm. *E*, quantification of the *nme3a* MO rescue experiment. Coinjection of *NME3* mRNA reduced the difference in tubular length between the *nme3a*-depleted and the uninjected side in *Xenopus* embryos (***, *p* < 0.001; *t* test; *error bars* represent S.D.).

The close biochemical association with known NPHPs and the previously reported function of NME family members in ciliary biogenesis prompted us to analyze the function of NME3 in two different vertebrate model systems, zebrafish and *Xenopus laevis*. First, the expression pattern of *nme3* was determined by *in situ* hybridization at different embryonic stages. In *Xenopus* as well as in zebrafish, the expression was ubiquitous with above average expression in the central nervous system and the developing kidney in *Xenopus* embryos (Fig. S2, *A* and *B*). For loss-of-function experiments, we designed two individual translation-blocking antisense morpholino oligonucleotides (MOs) targeting the two different homeologs that exist for *nme3* in *Xenopus*. The injection of either MO alone or a combination of both did not affect the gross morphology of *nme3* morphants (Fig. S2*C*) but had pronounced effects on the elongation of the embryonic renal tubule (Fig. 3, *A* and *B*). The strongest phenotype was observed by knockdown of *nme3a* alone. Addition of *nme3b* MO did not decrease tubule length further, indicating that the presence of Nme3a is the rate-limiting factor and that Nme3b acts in addition, but not redundantly, to Nme3a (Fig. 3, *A* and *B*). The tubule-shortening phenotype has been observed as a result of knocking down a number of known NPHPs genes such as *invs*, *nphp3*, *nek8*, and *anks6*. Accordingly, *in situ* hybridization of different pronephric segment markers showed a result similar to that already seen after depletion of *nek8*, *nphp3*, and *invs* in *Xenopus* embryos (6, 32). *nme3a* morphants showed diminished expression of all pronephric segment markers, resulting in decreased tubular length (Fig. 3*C*). Rescue experiments of *nme3a* MO with human *NME3* mRNA reduced the severity of the tubule-shortening phenotype significantly and served as a specificity control of the *nme3* knockdown phenotype (Fig. 3, *D* and *E*).

**Figure 4.**
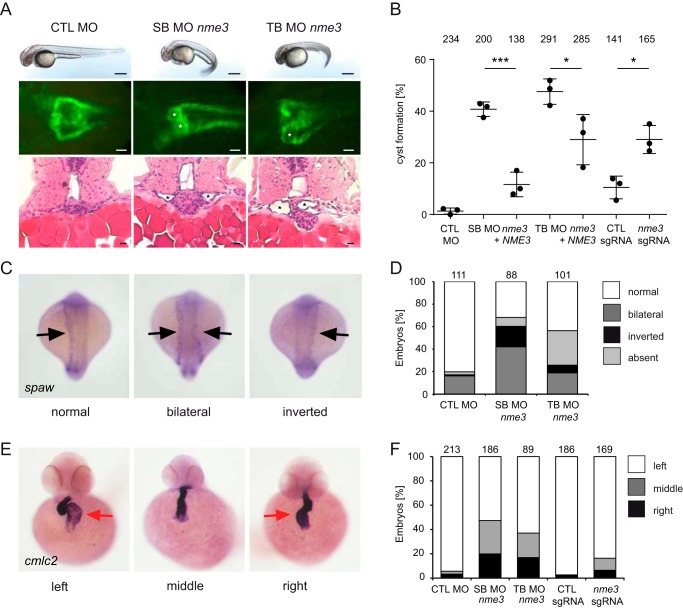
**Knockdown of *nme3* in zebrafish results in cyst formation and left-right asymmetry defects.** *A*, zebrafish embryos injected with control (*CTL*) MO, *nme3* SB MO, or *nme3* TB MO at 48 hpf. In contrast to the control embryos, *nme3* morphants showed a curly-tail phenotype. *Scale bars*, 100 μm. Nme3-depleted zebrafish embryos showed pronephric cyst formation, visualized using the transgenic *Tg(wt1b:EGFP)* line (*white asterisks*). *Scale bars*, 50 μm. Histological sections of *nme3* morphants stained by hematoxylin and eosin confirmed cyst formation (*black asterisks*). *Scale bars*, 10 μm. *B*, quantification of the percentage of cyst formation caused by knockdown and CRISPR-mediated mosaic indel formation of *nme3* at 48 hpf (*, *p* < 0.05; ***, *p* < 0.001; *t* test; *error bars* represent S.D.). *C*, representative pictures of 18-somite-stage zebrafish embryos after WISH for *spaw* with normal, bilateral, or inverted *spaw* expression (*black arrows*). *D*, quantification of the percentage of altered *spaw* expression in *nme3* zebrafish morphants. *E*, examples of zebrafish embryos with normal (*left*) and reversed (*middle* and *right*) heart looping, visualized by WISH for the heart-looping marker *cmlc2* at 48 hpf. The *red arrows* point to the atria. *F*, quantification of the percentage of heart laterality defects caused by *nme3* depletion in zebrafish embryos at 48 hpf.

In zebrafish, only a single gene copy of *NME3* exists. We designed two MOs, one translation-blocking MO and a second splice-blocking MO. Efficacy of the splice-blocking MO was confirmed by reverse transcription-PCR, which detected a transcript that was 66 bp (in exon 2) shorter than the WT as confirmed by sequencing (Fig. S2*D*). Because the resulting deletion of 22 amino acids is inside the catalytic NDPK domain, the remaining protein is expected to be functionally inactive. In about 40% of the zebrafish larvae, injection of either MO led to a ventrally curved body axis and the formation of pronephric cysts (Fig. 4*A*), similar to the phenotype that has been observed after knockdown of many ciliary and most NPH-associated genes (33, 34). The specificity of the knockdown phenotype was confirmed by coinjection of the human *NME3* mRNA, which rescued the frequency of cyst formation significantly (Fig. 4*B*). In addition, CRISPR-mediated indel formation in *nme3* resulted in a significant rate of cyst formation (Figs. 4*B* and S2E). Phenotypic analysis in *Xenopus* and zebrafish revealed that Nme3 is essential for normal development of the embryonic kidney. Some patients affected by NPH show extrarenal manifestations, including laterality defects (*e.g.* situs inversus), a phenotype also observed in animal models of NPH (35–37). We therefore tested whether laterality defects occurred in *nme3*-depleted zebrafish. In the 18-somite stage of WT embryos, the expression of *southpaw* (*spaw*) was asymmetrically stronger on the left side, whereas *nme3* deficiency resulted in an increased number of embryos either with stronger expression on the right side or equal expression (Fig. 4, *C* and *D*). One of the earliest manifestations of axis asymmetry in zebrafish is the turning of the heart tubule. In the majority of WT embryos, an L-type (left-looping) heart was detected by *in situ* hybridization for *cmlc2*, but knockdown of *nme3* and CRISPR-mediated mosaic indel formation resulted in an increase of reversed heart looping (Fig. 4, *E* and *F*). Hence, these data suggest that Nme3 is involved in establishing asymmetric laterality in zebrafish.

**Figure 5.**
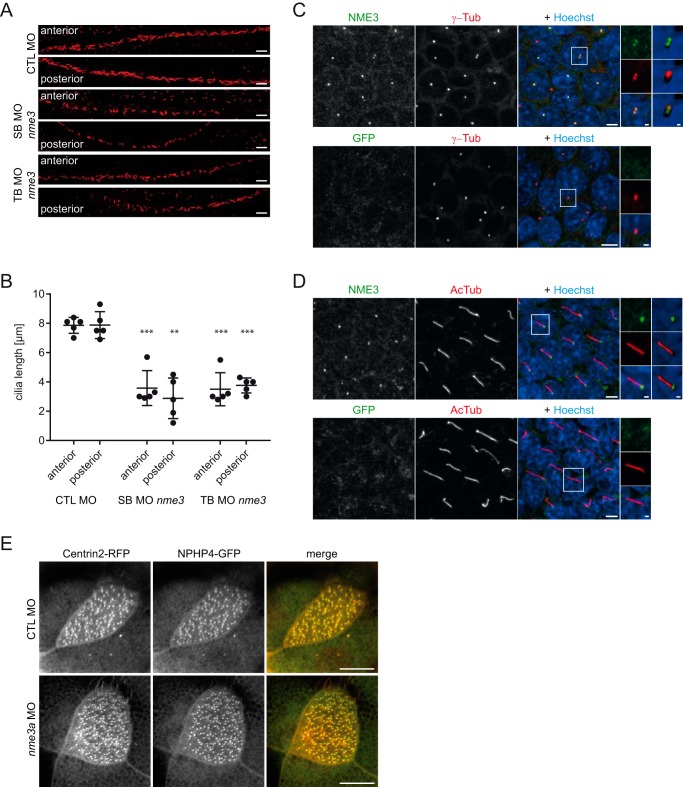
**Nme3 is important for ciliogenesis in zebrafish and localizes to the ciliary basal body of mIMCD3 cells.** *A*, confocal images of the anterior and posterior segments of the ciliated pronephric tubules of 24-hpf control (*CTL*) and *nme3*-deficient zebrafish embryos. Cilia were visualized by acetylated tubulin staining. *Scale bars*, 10 μm. *B*, quantification of the ciliary length in pronephric tubules. In contrast to the controls, ciliary length was affected by knockdown of *nme3* (**, *p* = 0.0063; ***, *p* < 0.001; *t* test, *error bars* represent S.D.). *C*, confocal images of 6-day-starved mIMCD3 cells stained with antibodies against γ-tubulin (γ-*Tub*) (centrosomal marker; *red*), NME3 (*green*), and Hoechst (DNA; *blue*). NME3 colocalizes with γ-tubulin at the centrioles of centrosomes. *D*, costaining of cilia by acetylated tubulin (*AcTub*) (ciliary axoneme; *red*) identifies NME3 at the basal body of primary cilia. Immunofluorescence staining with an anti-GFP antibody serves as a control and results in the absence of centrosome and basal body labeling (*bottom row*). *Scale bars*, 5 μm. Magnifications and three-dimensional reconstructions are shown in *white boxes. E*, NPHP4–GFP and Centrin–RFP were coexpressed in epidermal multiciliated cells of stage 30 *Xenopus* embryos and detected by confocal microscopy. NPHP4 colocalization with the basal body marker Centrin was not altered by injection of *nme3a* morpholino in at least 25 cells observed in three experiments. *Scale bars*, 1 (*C* and *D*) and 10 μm (*E*).

Laterality defects have been observed in animals defective in ciliary signaling and structure. In addition, pronephric cysts in zebrafish can occur as a result of the lack of cilia or the presence of malformed cilia in the pronephric tubule. To determine whether Nme3 depletion would impair ciliogenesis or the structural integrity of cilia, we measured the length of cilia in the zebrafish pronephric tubule. Although cilia were present, in *nme3* morphants they were significantly shorter (Fig. 5, *A* and *B*), demonstrating that Nme3 is crucial for ciliary extension or ciliary length control.

**Figure 6.**
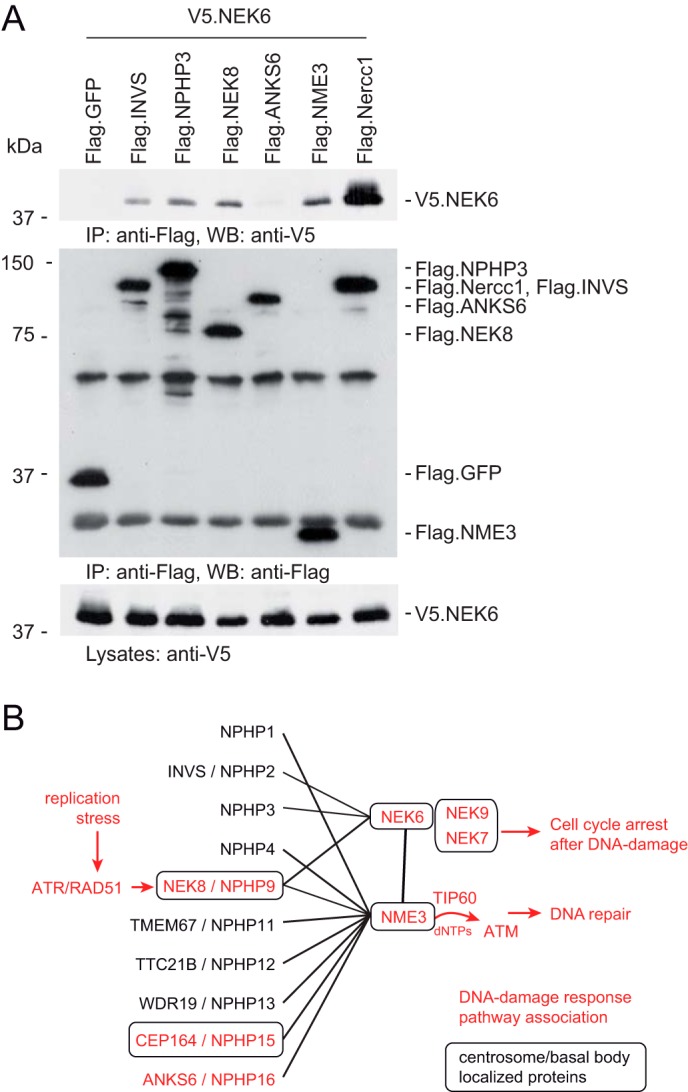
**NME3 and NEK6 interact with each other.** *A*, V5-tagged NEK6 was coexpressed with different FLAG-tagged proteins in HEK293T cells. After immunoprecipitation with an anti-FLAG antibody, NEK6 was detected in the precipitates of INVS, NPHP3, NME3, and NERCC1 (NEK9). *B*, schematic protein–protein interaction network. Basal body/centrosome-associated proteins are *boxed*, and proteins in *red* have been associated with the DNA-damage repair pathway. *IP*, immunoprecipitation; *WB*, Western blotting.

As the observed phenotypes strongly suggest a role for NME3 at the cilium, we determined NME3's subcellular localization. Because the available antibodies are directed against mouse Nme3, we performed immunofluorescence in fully confluent murine IMCD cells. Staining for NME3 revealed a colocalization with γ-tubulin at the basal bodies of ciliated mIMCD3 cells (Fig. 5, *C* and *D*). Thus, NME3 shares, with NPHPs such as NEK8 and CEP164 in quiescent renal cells, a subcellular localization at the basal body. Because NPHP4 has been shown to be localized at the basal bodies of multiciliated epidermal cells in *Xenopus* (38), we investigated whether Nme3 would be required for the correct localization of this protein. However, knockdown of *nme3a* did not affect the localization of NPHP4 at ciliary basal bodies (Fig. 5*E*). To gain further insight into the function of NME3, we sought to identify other NME3-associating proteins and used an unbiased protein interaction screen using FLAG-tagged NME3 as bait (full results are in Table S1). Among prey proteins associated with the cilium, we found NEK6 as an interaction partner of NME3 and confirmed its binding to NME3 by coimmunoprecipitation experiments in HEK293T cells (Fig. 6*A*). NEK6 is a member of the never in mitosis gene A (NIMA) kinase family and contains a conserved N-terminal kinase domain (serine/threonine kinases) (39). NEK6 is required for mitotic spindle formation and cytokinesis, promotes centrosome separation (40), is activated downstream of its interaction partner NEK9 (NERCC1), and is colocalized with the centrosome (41). In addition, NEK6 has also been implicated in DNA-damage control. It is a target of the DNA-damage checkpoint kinases Chd1 and dChk2, and inhibition of NEK6 is required for proper cell cycle arrest after DNA damage (42). Further analysis revealed that NEK6 is also a physical binding partner for INVS, NPHP3, and NEK8 (Fig. 6*A*). Consistent with this, in a recent yeast two-hybrid screen performed to characterize the NEK6 interactome, NPHP3 was identified as an interaction partner for NEK6 (41). In conclusion, NME3 closely associates with the centrosomal protein NEK6.

To determine the relevance of *NME3* as a potential novel ciliopathy gene, we performed targeted sequencing of all five coding exons and adjacent splice sites of *NME3* (GenBank^TM^ accession number NM_002513.2) in 768 individuals with NPH-related ciliopathies. We did not detect biallelic mutations in any of the included patients. However, we detected a heterozygous nonsense allele in exon 5 (c.477G→A, p.Trp159*) in two unrelated individuals, but no second variant could be identified. Therefore, under an autosomal recessive hypothesis, this finding is not sufficient to explain the phenotype.

## Discussion

The present study reveals a novel role for the nucleoside-diphosphate kinase NME3 in ciliary function. NME3 has recently been identified as a key supplier of dNTPs used in the DNA-repair process activated during replication stress (22). Two unbiased screens independently discovered that the nephronophthisis protein NEK8 stabilized components of the DNA-repair machinery at stalled replication forks (17, 19). We show that NME3 physically interacts with NEK8 and other NPHPs, some of which have also been implicated to act in the DNA-damage response (Fig. 6*B*). Similar to previous observations of NEK8 knockdown cells, we also detected increased nuclear γH2AX signals during aphidicolin-induced replication stress when ANKS6 was knocked down. Our data add NME3 and ANKS6 to the number of proteins linked to both ciliopathies and the DNA-damage response and thus strengthen substantially the notion that replication stress and the genomic instability of tubule cells may contribute to the pathogenesis of nephronophthisis (3). The precise functional relationships among the involved components and analysis of the remaining NPHPs for a potential role in genomic stability await further studies. Interestingly, depletion of the latest gene product identified to cause nephronophthisis (MAPKBP1) leads to increased DNA-damage response signaling, but MAPKBP1 has not been detected in the cilium or its appendages (43).

Non-Mendelian inheritance of a t-haplotype from male mice to their offspring is affected by Nme3 heterozygous deletion and is likely due to sperm motility defects (44). The targets of NME3 in male germ cells are elusive, and its functional role outside of spermatogenesis has not yet been assessed. Here, we show that NME3 physically interacts with multiple NPHPs and that the phenotype of *nme3* knockdown in zebrafish and *Xenopus* is similar to previously published phenotypes of known NPHPs. In addition, the detection of endogenous NME3 at the base of the cilium is consistent with a role in ciliary function. Hence, our data provide evidence that *NME3* represents a potential nephronophthisis disease gene.

NME3 not only physically interacts with several NPHPs but is also localized at the basal bodies of primary cilia where the interaction with NEK8 and CEP164 may take place. The interaction of NME3 with other NPHPs that have not been detected at the basal body (*e.g.* ANKS6, WDR19, and TTC21B) could depend on intermediaries (*e.g.* NEK8), which can move between multiple ciliary compartments (5, 14). A second possibility is that NME3 interacts with proteins as they are shuttled through the basal body, which serves as an entry point for nonmembranous proteins into the cilium (45). The centrosomal protein CEP290 (NPHP6) can act as a ciliary gatekeeper where it regulates the trafficking of proteins between the proximal cilium and the transition zone (46). One such example is NPHP3, which passes through the basal body on its passage to the cilium (47). A high-throughput interaction screen (BioPlex 2.0) recently identified six components of the BBSome (BBS2, BBS4, BBS5, BBS7, TTC8/BBS8, and BBS9) that regulate dynamic protein transport into the cilium as potential NME3 interactors (48). Further experimental work is needed to examine a potential role for NME3 in controlling the transport of NPHPs in and out of the cilium (49).

NME3 belongs to a subgroup of NDPKs with strong kinase activity (group I; NME1–4) that provides NTPs such as GTP as a cofactor for heterotrimeric G-proteins. NME3 represents a good candidate to provide the substrate for ciliary GTPases.

NME3 could provide the substrate for cilium-associated G-proteins such as RPGR (retinitis pigmentosa (50)), GPR48 (polycystic kidneys (51)), RanGDP/GTP (52), and ARL6 (retinitis pigmentosa and BBS (53)). IFT22 and IFT27 (BBS19) are involved in retrograde intraflagellar transport in *Trypanosoma brucei* and have a GTPase domain (54). In addition, should a genetically encoded sensor that could be targeted to the cilium become available, it would be interesting to directly determine whether GTP concentrations in cilia depend on NME3.

## Experimental procedures

### Xenopus and zebrafish injection and manipulation

*X. laevis* embryos were cultured, manipulated, and staged as described before (32). In short, females were injected with 800 units of human chorionic gonadotropin the day before harvesting eggs. *In vitro* fertilized embryos were cultured in 0.3× Marc's modified Ringer's medium, and injections were performed in 3% Ficoll in 0.3× Marc's modified Ringer's medium.

Microinjection of 10 nl into the ventrolateral-vegetal blastomeres of *Xenopus* was performed to target the pronephros anlage at the four- to eight-cell stage. RFP mRNA was coinjected as an injection control, and only fluorescent embryos were used for further analysis.

Zebrafish (strain *Tg(wt1b:EGFP)*) (55) were bred and maintained under standard conditions at 28.5 °C. Zebrafish injection experiments were performed as described previously (56). Briefly, morpholinos and sense RNA were diluted in 0.1 m KCl to concentrations of 6 μg/μl and 20 ng/μl, respectively. One nanoliter of these dilutions was injected through the chorion of one- or two-cell stage embryos. The sequence of antisense oligonucleotide translation/splice-blocking (TB/SB) MOs (GeneTools, LLC) used for targeted knockdown were as follows: zf SB-MO *nme3* (6 ng), 5′-ATGCTGTATTAGGGTTCTCACCTGC-3′; zf TB-MO *nme3* (6 ng), 5′-GATCATCTTCTCCGTGCAGGAATCC-3′; xl TB *nme3a* MO (16 ng), 5′-AGCACCAGGCAGATCATGGCGAGAC-3′; xl TB *nme3b* MO (16 ng), 5′-GCACCAGACAGATCATGGCGAGAGC-3′.

For CRISPR-mediated mosaic indel formation in the F0 generation, a single guide RNA (sgRNA) targeting the sequence GGATGGAGTTCAGCGCAGACTGG (Protospacer adjacent motif sequence underlined) was generated by reverse transcription by T7 polymerase. One nanoliter of injection mixture containing *nme3* sgRNA (50 ng/μl) together with recombinant Cas9 protein (PNA-Bio; 300 ng/μl) was injected into one-cell stage embryos.

To confirm successful indel formation, the genomic DNA was amplified using the primers znme3_gRNA_ID_F (5′-CGTAAGGATTCACTGCTGTTTT-3′) and znme3_gRNA_ID_R (5′-GCATGAGTTGTTTTCCATTGTCA-3′). Sanger-sequenced PCR products were analyzed using the inference of CRISPR edits from Sanger trace data (ICE) method (57).

*In vitro* synthesis of mRNA was performed using an mMESSAGE mMACHINE kit (Ambion) using T7 polymerase. Plasmid linearization was performed with SalI (VF10GFP-NPHP4; VF10-nme3) or NotI (Centrin2-RFP). Multiciliated epidermal cells were imaged using a Carl Zeiss LSM 880 Observer microscope using the Fast AiryScan mode. Image processing was done in ZEN-Blue and ImageJ. All animal experiments were approved by the institution's Animal Committee (Regierungspräsidium Baden-Württemberg).

### RNA extraction and RT-PCR

RNA was isolated from zebrafish embryos following the RNeasy manual (Qiagen), and cDNA synthesis was performed using the ProtoScript First Strand cDNA Synthesis kit (32). For RT-PCR, the following gene-specific primers were used: z*nme*3_forward, 5′-ATTCCTGCACGGAGAAGATG-3′; z*nme*3_reverse, 5′-CTCGTGCAGAGCTGACTTTG-3′. For eukaryotic translation elongation factor1α (ef1α), the following primers were used: z*ef1*α_forward, 5′-ATCTACAAATGCGGTGGAAT-3′; z*ef1*α_reverse, 5′-ATACCAGCCTCAAACTCACC-3′.

### Cloning and plasmids

Full-length NME3 was subcloned from NME3pDONR223 clone (BC000250) into V5- and FLAG-tagged pcDNA6 vector (Invitrogen) and for *in vitro* transcription into VF10. A complete coding sequence of ARL13B was cloned from an ARL13B cDNA clone (MGC:120611 (IMAGE:40026398), Source BioScience). ANKRD13A was cloned from a cDNA clone (MGC:26764 (IMAGE:4830174), Source BioScience) and further subcloned into V5- and FLAG-tagged pcDNA6 vector. Full-length NEK6 was amplified from 293T cDNA using the primers 5′-CGCGGGACGCGTATGCCCAGGAGAGAAGTT-3′ and 5′-GAATGCGGCCGCTCAGGTGCTGGACATCCAGATGT-3′ and afterward subcloned into V5- and FLAG-tagged pcDNA6 vector (Invitrogen) via the MluI and NotI cloning sites. Full-length *nme3a* and *nme3b* were amplified from *Xenopus* cDNA with the following primers: xlNme3a_mlu_for, 5′-CGCGGGACGCGTATGATCTGCCTGGTGCTCACCATC-3′; xlNme3a_not_rev, 5′-GAATGCGGCCGCTTATTCATATATCCAGCTCTCAGCAC-3′; xlNme3b_mlu_for, 5′-CGCGGGACGCGTATGATCTGTCTGGTGCTCACCATCT-3′; xlNme3b_not_rev, 5′-GAATGCGGCCGCTTATTCATATATCCATCTCT-3′. PCR products were subcloned into pGEM-T vector (Promega).

Cloning of the template plasmid for sgRNA synthesis was performed using the primers znme3_gRNA_F (5′-TAGGGGATGGAGTTCAGCGCAGAC-3′) and znme3_gRNA_R (5′-AAACGTCTGCGCTGAACTCCATCC-3′) into pT7-gRNA vector. pT7tyrgRNA was used to generate the control sgRNA and was a gift from Wenbiao Chen (Addgene plasmid number 46761) (42).

### Whole-mount in situ hybridization and immunostaining

Whole-mount *in situ* hybridization (WISH) was performed with digoxigenin-labeled antisense probes as described before (32, 58). For synthesis of *in situ* probes, plasmids were linearized, and digoxigenin-labeled RNA was transcribed using an SP6 or T7 digoxigenin labeling kit (Roche Applied Science). *nme3* antisense probe for zebrafish was synthesized from EcoRI-linearized zNME3_pSPORT1 plasmid (expressed sequence tag clone SUUkp6110A03137Q, Source BioScience) using SP6. *Xenopus nme3* sense probes were transcribed by T7 polymerase from nme3a/b_pGEM-T SalI-linearized plasmids. For synthesis of antisense probes, SacII-linearized plasmids were transcribed with SP6. Anti-digoxigenin antibody conjugated to alkaline phosphatase was used to detect bound probes (Roche Applied Science). For *Xenopus* rescue experiments, whole embryos were stained with fluorescein-conjugated *Lycopersicon esculentum* lectin (Vector Laboratories). The length of the pronephros was measured using ImageJ, and afterward the difference in kidney length between the uninjected and the MO-injected side was calculated. *cmlc2* WISH was used for 48-h-postfertilization (hpf) zebrafish embryos, and *spaw* WISH was used for 18-somite-stage embryos. Zebrafish whole-mount antibody staining was performed according to standard procedures. Briefly, zebrafish embryos were fixed in 4% paraformaldehyde, 1% DMSO overnight at 4 °C; equilibrated in 100% MeOH at −20 °C for 1 h; Proteinase K–digested (10 μg/ml) for 20 min; treated with ice-cold acetone for 5 min at −20 °C; incubated in blocking solution (PBS with 0.1% Tween 20 and 0.1% Triton X-100, 1% DMSO, 2% sheep serum, 1% BSA); and then incubated with anti-acetylated tubulin (Sigma-Aldrich, T6793; 1:500) and Cy3-labeled secondary antibody (Jackson ImmunoResearch Laboratories, 715-165-150; 1:1000). Confocal images were generated with a Carl Zeiss LSM510 laser-scanning microscope. Confocal z-stacks were projected to one plane (maximum intensity projection). The length of clearly distinguishable single cilia was measured using ImageJ as described previously (59).

### Histology and histochemistry

Zebrafish embryos were embedded in Technovit 7100 (Heraeus), sectioned on a microtome (Leica), stained with hematoxylin and eosin, and imaged with an Axioplan2 (Carl Zeiss) microscope and AxioVision software (Carl Zeiss).

### Cell culture and transfection

HEK293T cells were cultured in Dulbecco's modified Eagle's medium with 10% fetal bovine serum. mIMCD3 cells were cultured in Dulbecco's modified Eagle's medium–F-12 medium with 10% fetal bovine serum. HEK293T cells were transfected using the calcium phosphate method.

### Coimmunoprecipitation and Western blotting

After 24 h of transfection, HEK293T cells were washed with PBS and lysed (1% Triton X-100, 20 mm Tris, pH 7.5, 50 mm NaCl, 50 mm NaF, 15 mm Na_4_P_2_O_7_, 0.1 mm EDTA). The lysates were supplemented with 2 mm Na_3_VO_4_ and protease inhibitor mixture (Roche Applied Science), and after ultracentrifugation the lysates were incubated with anti-FLAG M2 agarose affinity M2 beads for 2 h and then washed with lysis buffer. Proteins were fractionated by SDS-PAGE, and the following primary antibodies were used for protein detection by Western blot analysis: mouse anti-FLAG (Sigma-Aldrich, F3165) and rabbit anti-V5 (Millipore, AB3792).

### Immunofluorescence staining and confocal imaging of cilia in mIMCD3 cells

For the ciliogenesis assay, mIMCD3 cells were grown on glass coverslips for 6 days. After methanol fixation, the cells were stained with the primary antibodies rabbit anti-NME3 (Proteintech, 15136-1-AP; 1:100), -acetylated tubulin (Sigma-Aldrich, T6793; 1:2000), and -γ-tubulin (Sigma-Aldrich, T6557; 1:200) and with Hoechst 33342. Antibodies were visualized using Cy3- or Alexa Fluor–488-labeled secondary antibodies at a dilution of 1:500 (Jackson ImmunoResearch Laboratories). Confocal microscopy was done using the LSM510 Duo-Live microscope equipped with an LCI Plan-Neofluar 63×/1.3 Imm Korr DIC (differential interference contrast) objective (Carl Zeiss). ZEN-Black 2010 software (Carl Zeiss) was used for image acquisition. z-stacks were taken to include all cilia in different z-positions and then projected to one plane (maximum intensity projection). To study three-dimensional (3D) protein distributions, z-stacks were converted to 3D images in Imaris 7.6 (Bitplane).

### γH2AX staining and high-content screening microscopy

mIMCD3 cells expressing an shRNA construct targeting Anks6 or luciferase (negative control) have been described previously (6). Cells were grown in the presence or absence of 0.125 μg/ml tetracycline for at least 6 days, plated on glass-bottom multiwell plates, and incubated with or without 800 nm aphidicolin for 24 h under serum starvation. Immunostaining was performed after paraformaldehyde fixation using a primary antibody against phosphohistone H2A.X (Ser-139) (Cell Signaling Technology, 9718; 1:500) and a Cy3-labeled secondary antibody (Jackson ImmunoResearch Laboratories, Dianova, 711-165-152; 1:500). High-content screening was performed using the Olympus Scan^R^ imaging and analysis platform with a 20× LUCPLFLN 0.45 numerical aperture objective. Violin plots were generated using R and R-Studio.

### Affinity proteomics

For FLAG immunoprecipitation, washed immunoprecipitates were incubated with FLAG peptide (Sigma) to remove the precipitated protein from the anti-FLAG–coated beads. Samples were reduced by 1 m DTT for 5 min at 95 °C and alkylated with 5.5 mm iodoacetamide for 30 min at 25 °C. After separation on an SDS gel, the lanes were cut into 10 slices, and in-gel digestion was performed using trypsin. Peptides were extracted from the gel slices using 5% formic acid. The peptides in samples of extracts were separated by HPLC using either an Agilent 1200 nanoflow HPLC (Agilent Technologies GmbH, Waldbronn, Germany) or an Eksigent NanoLC-ultra-HPLC using linear gradients from 10 to 30% buffer B (0.1% v/v formic acid in acetonitrile) at a flow rate of 250 nl/min and examined with an LTQ Orbitrap XL mass spectrometer (Thermo Fisher Scientific, Bremen, Germany) that was coupled to the HPLC. The MS raw data files were uploaded into the MaxQuant software (1.4.1.2) and further condensed for the samples (experiment and control) in Scaffold4 (Proteome Software, Portland, OR).

### Mutational analysis

768 individuals with renal ciliopathies (NPHP-RC) were recruited worldwide. Pedigree information was obtained and was compatible with a recessive mode of inheritance. The suspected diagnosis of NPHP-RC was based on clinical criteria such as increased echogenicity and/or cysts observed by renal ultrasound. Approval for human subject research was obtained from the Institutional Review Board committees of the University of Michigan and of Boston Children's Hospital. All analyses abide by the Declaration of Helsinki principles. Pathogenic mutations in any of the 13 most frequent monogenic genes of renal ciliopathies were excluded prior to the study (60). Genomic DNA was extracted from peripheral blood samples by standard salt-precipitation methods. Targeted amplification was performed by multiplexed PCR using Fluidigm Access-Array^TM^ technology followed by barcoding and next-generation resequencing on an Illumina^TM^ MiSeq platform as established previously by our group (60). Sanger DNA sequencing was further conducted for single-mutation confirmation. All coding exons and adjacent splice sites of *NME3* (GenBank accession number NM_002513.2) were analyzed.

### Statistical analysis

SigmaStat software was used to analyze statistical significance. All experiments shown were performed independently three times, and diagrams show the mean ± S.D. Tests used to calculate the significance are indicated in the corresponding figure legends for the individual experiments. The number of analyzed embryos is given above the bars.

## Author contributions

S. H., G. W., and S. S. L. conceptualization; S. H., D. E., N. F., S. S., D. A. B., J. H., F. H., and A. K.-Z. investigation; S. H., D. E., and S. S. L. writing-original draft; D. A. B., J. H., F. H., A. K.-Z., and S. S. L. formal analysis; A. K.-Z., C. B., G. W., and S. S. L. supervision; C. B. resources.

## Supplementary Material

Supporting Information

## Footnotes

4 The abbreviations used are:

NPH: nephronophthisis
NPHP: NPH protein
NEK: NIMA-related kinase
NIMA: never in mitosis gene A
CEP164: centrosomal protein 164
NME3: NME/NM23 nucleoside-diphosphate kinase 3
ANKS6: ankyrin repeat and sterile α motif domain–containing 6
BBS: Bardet–Biedl syndrome
IFT: intraflagellar transport
INVS: inversin
ATM: ataxia telangiectasia mutated
NDPK: nucleoside-diphosphate kinase
HEK: human embryonic kidney
IMPG: interphotoreceptor matrix glycoprotein
MO: morpholino oligonucleotide
IMCD: inner medullary collecting duct
mIMCD: murine IMCD
RFP: red fluorescent protein
TB: translation-blocking
SB: splice-blocking
sgRNA: single guide RNA
WISH: whole-mount *in situ* hybridization
hpf: hours postfertilization
RC: renal ciliopathies
shRNA: short hairpin RNA
*spaw*: *southpaw*.
