# Supplementary material for: The nucleoside-diphosphate kinase NME3 associates with nephronophthisis proteins and is required for ciliary function during renal development
Source: J Biol Chem. 2018 Aug 15;293(39):15243–55. doi: 10.1074/jbc.RA117.000847 (PMC6166740; doi:10.1074/jbc.RA117.000847)
Supplement: Supporting Information [file supp_293_39_15243__index.html]

The nucleoside-diphosphate kinase NME3 associates with nephronophthisis proteins and is required for ciliary function during renal development — Ciliary function of NME3 — Supporting Information 

# The nucleoside-diphosphate kinase NME3 associates with nephronophthisis proteins and is required for ciliary function during renal development

## Supporting Information

- Supporting Information (to be published online) - Supporting information
- Supporting Information (to be published online) - Supplementary Table 1 Results of the affinity purification experiment. The full list of detected proteins in two replicates is shown.
