# Supplementary material for: The nucleoside-diphosphate kinase NME3 associates with nephronophthisis proteins and is required for ciliary function during renal development
Source: J Biol Chem. 2018 Aug 15;293(39):15243–55. doi: 10.1074/jbc.RA117.000847 (PMC6166740; doi:10.1074/jbc.RA117.000847)
Supplement: Supporting Information [file supp_RA117.000847_133748_2_supp_180946_pdsd0n.pdf]

Supplementary Figure 1

A

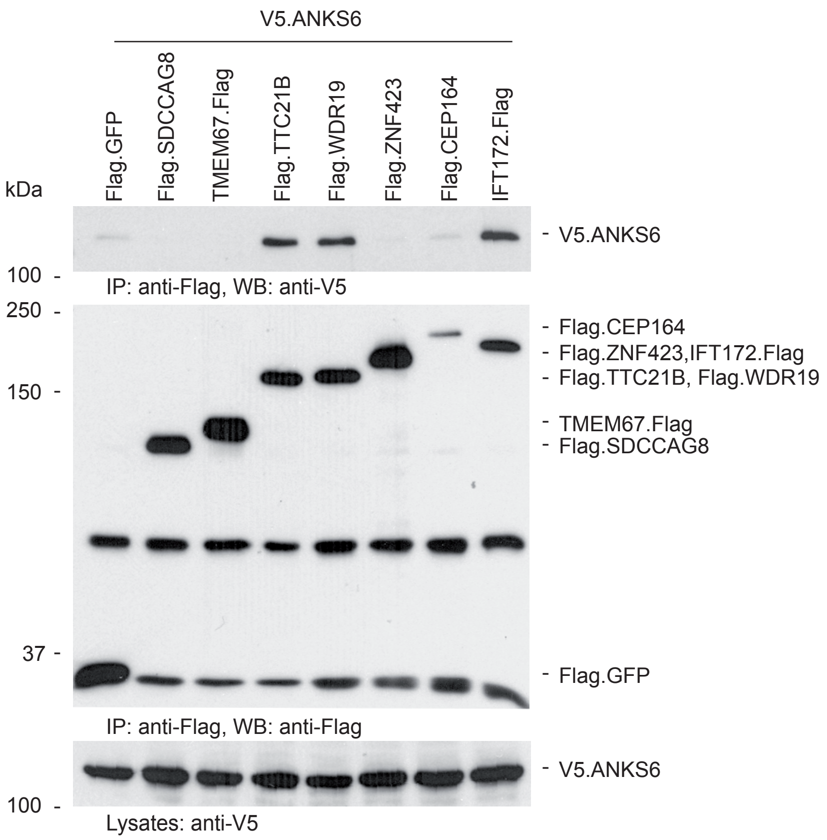

B

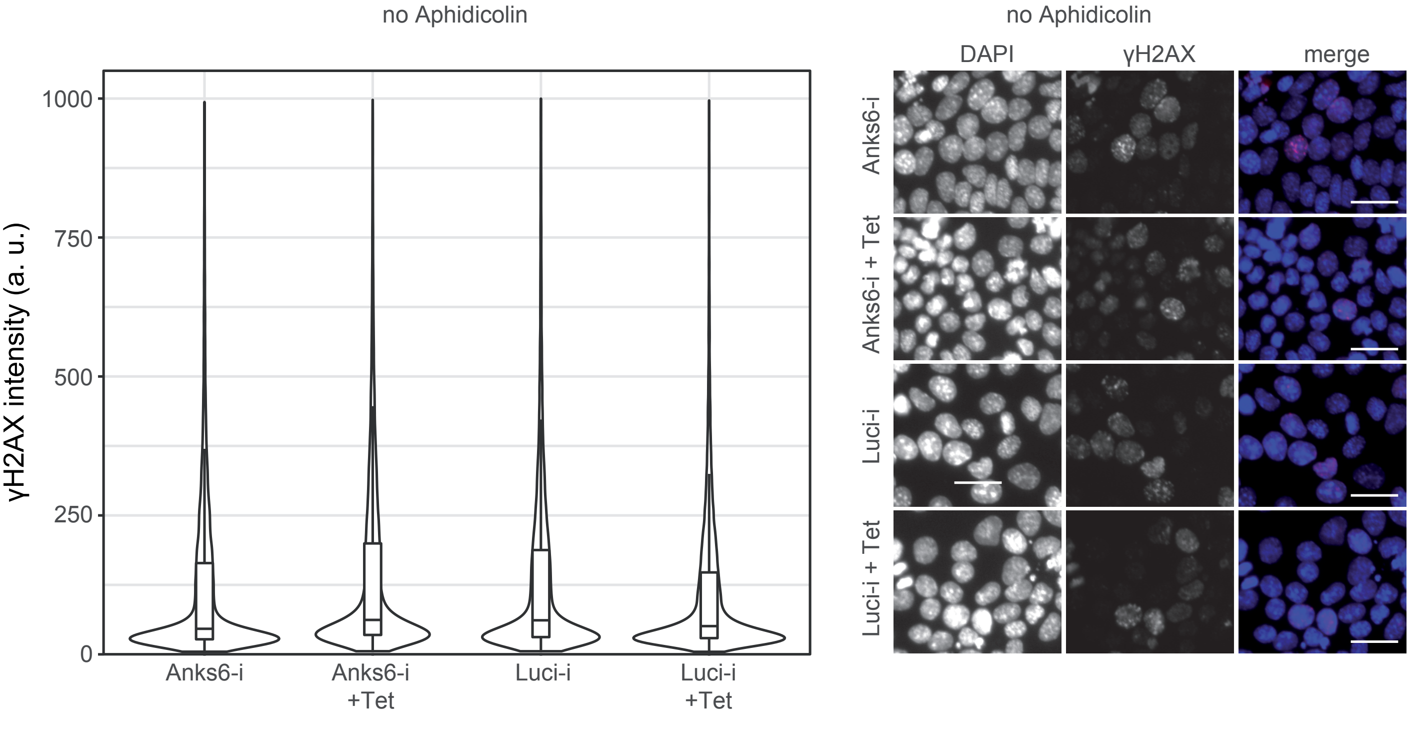

Supplementary Figure 2

A

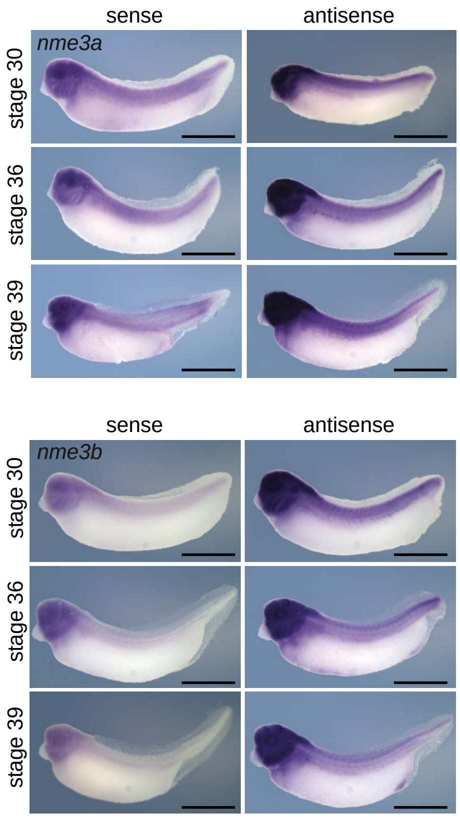

B

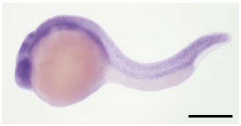

C

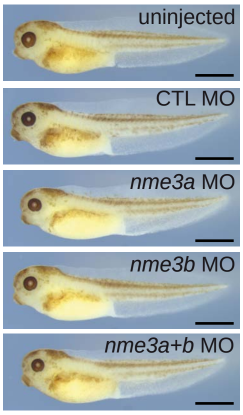

D

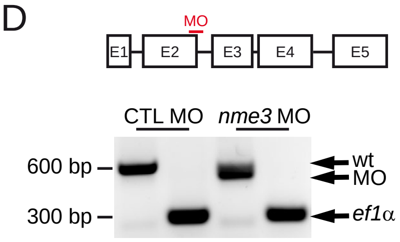

E

Indel percentage: 25%
knockout score: 21%

<table>
<tr><th>Indel frequency</th><th>base change</th><th>sequence</th></tr>
<tr><td>0.7354</td><td>0</td><td>GTGAAGCCGGATGGAGTTCAGCGCA|GACTGGTGGGTGAAATCATCCGCCGATTCGAAC</td></tr>
<tr><td>0.0747</td><td>+1</td><td>GTGAAGCCGGATGGAGTTCAGCGCA|nGACTGGTGGGTGAAATCATCCGCCGATTCGAA</td></tr>
<tr><td>0.0498</td><td>-4</td><td>GTGAAGCCGGATGGAGTTCAGCG--|--CTGGTGGGTGAAATCATCCGCCGATTCGAAC</td></tr>
<tr><td>0.0389</td><td>-5</td><td>GTGAAGCCGGATGGAGTTCAG----|-ACTGGTGGGTGAAATCATCCGCCGATTCGAAC</td></tr>
<tr><td>0.0150</td><td>-3</td><td>GTGAAGCCGGATGGAGTTCAGCGC-|--CTGGTGGGTGAAATCATCCGCCGATTCGAAC</td></tr>
<tr><td>0.0138</td><td>-5</td><td>GTGAAGCCGGATGGAGTTCAGCG--|---TGGTGGGTGAAATCATCCGCCGATTCGAAC</td></tr>
<tr><td>0.0124</td><td>-4</td><td>GTGAAGCCGGATGGAGTTCAGC---|-ACTGGTGGGTGAAATCATCCGCCGATTCGAAC</td></tr>
<tr><td>0.0113</td><td>-9</td><td>GTGAAGCCGGATGGAGTTCAGC---|------TGGGTGAAATCATCCGCCGATTCGAAC</td></tr>
<tr><td>0.0081</td><td>-7</td><td>GTGAAGCCGGATGGAGTTC------|-ACTGGTGGGTGAAATCATCCGCCGATTCGAAC</td></tr>
<tr><td>0.0046</td><td>+10</td><td>GTGAAGCCGGATGGAGTTCAGCGCA|nnnnnnnnnnGACTGGTGGGTGAAATCATCCGC</td></tr>
<tr><td>0.0045</td><td>-21</td><td>GTGAAGCCGGATGGA----------|------------GAAATCATCCGCCGATTCGAAC</td></tr>
<tr><td>0.0043</td><td>-7</td><td>GTGAAGCCGGATGGAGTT-------|GACTGGTGGGTGAAATCATCCGCCGATTCGAAC</td></tr>
<tr><td>0.0041</td><td>-23</td><td>GTGAAGCCGGATG------------|------------GAAATCATCCGCCGATTCGAAC</td></tr>
<tr><td>0.0037</td><td>+11</td><td>GTGAAGCCGGATGGAGTTCAGCGCA|nnnnnnnnnnnGACTGGTGGGTGAAATCATCCG</td></tr>
<tr><td>0.0016</td><td>-6</td><td>GTGAAGCCGGATGGAGTTCA-----|-ACTGGTGGGTGAAATCATCCGCCGATTCGAAC</td></tr>
<tr><td>0.0013</td><td>-6</td><td>GTGAAGCCGGATGGAGTTCAGC---|---TGGTGGGTGAAATCATCCGCCGATTCGAAC</td></tr>
<tr><td>0.0005</td><td>-9</td><td>GTGAAGCCGGATGGAG---------|GACTGGTGGGTGAAATCATCCGCCGATTCGAAC</td></tr>
<tr><td>0.0003</td><td>-23</td><td>GTGAAGCCGGA--------------|----------GTGAAATCATCCGCCGATTCGAAC</td></tr>
<tr><td>0.0003</td><td>-22</td><td>GTGAAGCCGGATGG-----------|------------GAAATCATCCGCCGATTCGAAC</td></tr>
</table>

Supplementary Figure 1

A) Transient overexpression of V5 tagged ANKS6 and Flag tagged NPHP proteins followed by immunoprecipitation with an anti-Flag antibody. ANKS6 was present in precipitates of TTC21B, WDR19 and IFT172. **B)** Intensity measurements of γH2AX immunostained IMCD3 cells in the absence of aphidicolin. At least 3000 cells were imaged and analyzed by high content screening microscopy and the mean intensity per cell is depicted in a violin plot. Box plots indicate the mean intensity per cell, and representative images of each condition are shown. Scale bars, 25µm (B).

Supplementary Figure 2

A) Lateral views of *Xenopus* embryos after WISH against *nme3a* and *nme3b* at indicated stages. Negative controls with the sense probe. *Nme3a* and *nme3b* showed a strong expression in the central nervous system and a weaker expression in the pronephros. Scale bars, 1 mm. **B)** Expression of *nme3* in zebrafish was examined by WISH. At 24 hpf *nme3* was ubiquitously expressed. Scale bar, 200µm. **C)** Representative pictures of characteristic *nme3 Xenopus* morphants of stage 40. Gross morphology was not affected by *nme3* depletion compared to the controls. Scale bars, 1mm. **D)** Schematic structure of the zebrafish *nme3* gene. The black boxes represent the exons and the lines in between the intron regions. The splice blocking morpholino (MO) used for the loss of function studies in zebrafish acts by targeting the splice donor site at the exon/intron boundary. The efficacy of the SB MO was confirmed by RT-PCR. The resulting PCR product of RNA prepared from *nme3* morphants was smaller compared to the wildtype product. Sequencing confirmed a loss of 66bp in exon2. The housekeeping gene *ef1α* served as a positive control. E) Result of the Inference of CRISPR Edits from Sanger Trace Data (ICE) analysis of *nme3* targeting sgRNA and Cas9 injected embryos. The knockout score of 21% indicates deleterious frameshift edits. The sgRNA target sequence is shown in blue, the PAM sequence is highlighted in red.

Supplementary Table 1

Results of the affinity purification experiment. The full list of detected proteins in two replicates is shown.
